# Phenotype-driven identification of epithelial signalling clusters

**DOI:** 10.1038/s41598-018-22293-x

**Published:** 2018-03-05

**Authors:** Elsa Marques, Tomi Peltola, Samuel Kaski, Juha Klefström

**Affiliations:** 1 Cancer Cell Circuitry Laboratory, Research Programs Unit/Translational Cancer Biology & Medicum, University of Helsinki, P.O Box 63 (street address: Haartmaninkatu 8), 00014 University of Helsinki, Helsinki, Finland; 2 Helsinki Institute for Information Technology HIIT, Department of Computer Science, Aalto University, PO BOX 15400 FI-00076 Aalto, Finland

## Abstract

In metazoans, epithelial architecture provides a context that dynamically modulates most if not all epithelial cell responses to intrinsic and extrinsic signals, including growth or survival signalling and transforming oncogene action. Three-dimensional (3D) epithelial culture systems provide tractable models to interrogate the function of human genetic determinants in establishment of context-dependency. We performed an arrayed genetic shRNA screen in mammary epithelial 3D cultures to identify new determinants of epithelial architecture, finding that the key phenotype impacting shRNAs altered not only the data population average but even more noticeably the population distribution. The broad distributions were attributable to sporadic gene silencing actions by shRNA in unselected populations. We employed Maximum Mean Discrepancy concept to capture similar population distribution patterns and demonstrate here the feasibility of the test in identifying an impact of shRNA in populations of 3D structures. Integration of the clustered morphometric data with protein-protein interactions data enabled hypothesis generation of novel biological pathways underlying similar 3D phenotype alterations. The results present a new strategy for 3D phenotype-driven pathway analysis, which is expected to accelerate discovery of context-dependent gene functions in epithelial biology and tumorigenesis.

## Introduction

Epithelial cells form sheets that line the body cavities, surfaces of different organs and they also form specialized glandular structures. Characteristic for epithelial cells is that they jointly respond to external and internal stimuli to deliver epithelial-specific functions such as directional secretion. Such tissue-wide actions require well-developed communication systems across the layers of epithelial cells, to ensure that the whole tissue performs or responds in a unified manner^1^. The joint specialized signalling functions of the apicobasally polarized epithelial cells are partly coordinated via the same specialized cell-cell junctions that establish the structural cohesion; tight junctions, adhesion junctions, desmosomes and the cell-basement membrane (BM) connecting hemidesmosomes^2,3^. In cancer, loss of organized epithelial structure is the most important diagnostic criterion and a hallmark of progression^4,5^. Interestingly, emerging evidence suggests that loss of epithelial integrity is not just an epiphenomenon in cancer, but that loss of proper epithelial cell interactions with each other and the extracellular matrix actually contributes to perturbed cell growth, metabolism and proliferation programs as well as the invasiveness of cancerous cells^6–9^. While it is now clear that the structure and organization of epithelial tissue (*context*) fundamentally regulates individual cell functions within the epithelium and that loss of these control mechanisms contributes to neoplasia, little is still known how that secondary level signalling controls the biology of epithelial cells^10^. Three–dimensional (3D) culture models of BM-attached epithelial tissues, pioneered by Mina Bissell and collaborators, have greatly facilitated studies aiming to clarify the context-dependencies of epithelial cell functions in health and disease^11,12^. The 3D cultures are amenable for both genetic and pharmacological perturbation and the perturbed epithelial phenotypes and functions can be comprehensively visualized with confocal fluorescence microscopy or other imaging tools^13–16^.

However, while genetic perturbation of such 3D structures that develop from single cells is relatively straightforward by shRNA or gene editing tools, the statistical population level analysis of perturbed 3D phenotypes can be challenging. The low throughput of phenotype analysis can present a major bottleneck in genetic screens, which produce large image datasets. Currently, there are no standard procedures for systematic analysis of genetically perturbed populations of epithelial 3D structures.

Here we present a new statistical framework for systematic analysis of the shRNA mediated phenotypic effects in arrayed populations of MCF10A 3D acinar structures. The statistical framework is based on univariate non-parametric Wilcoxon Rank-Sum statistical test and multivariate maximum mean discrepancy (MMD) analysis of the population distributions^17,18^. The MMD test metrics was employed to describe the population distances in form of heatmap and network visualization. The statistical significance defined phenotype-linked MMD networks were then integrated with a STRING database of protein-protein interactions. These integrated phenotype-proteome networks exposed protein-protein interactions known to be critical for the formation of epithelial morphology as well as new hypothetical protein networks. We also provide evidence suggesting that MYC oncoprotein-dependent changes in epithelial integrity can be interrogated through phenotype associated protein networks. In broader scope, the Wilcoxon-MMD statistical framework offers a robust data analysis platform suitable for analysis of any genetic or pharmacological screen with 3D phenotype change as a read-out.

## Results

### Impact of a loss-of gene function to 3D epithelial structures in three-dimensional culture

**Figure 1:**
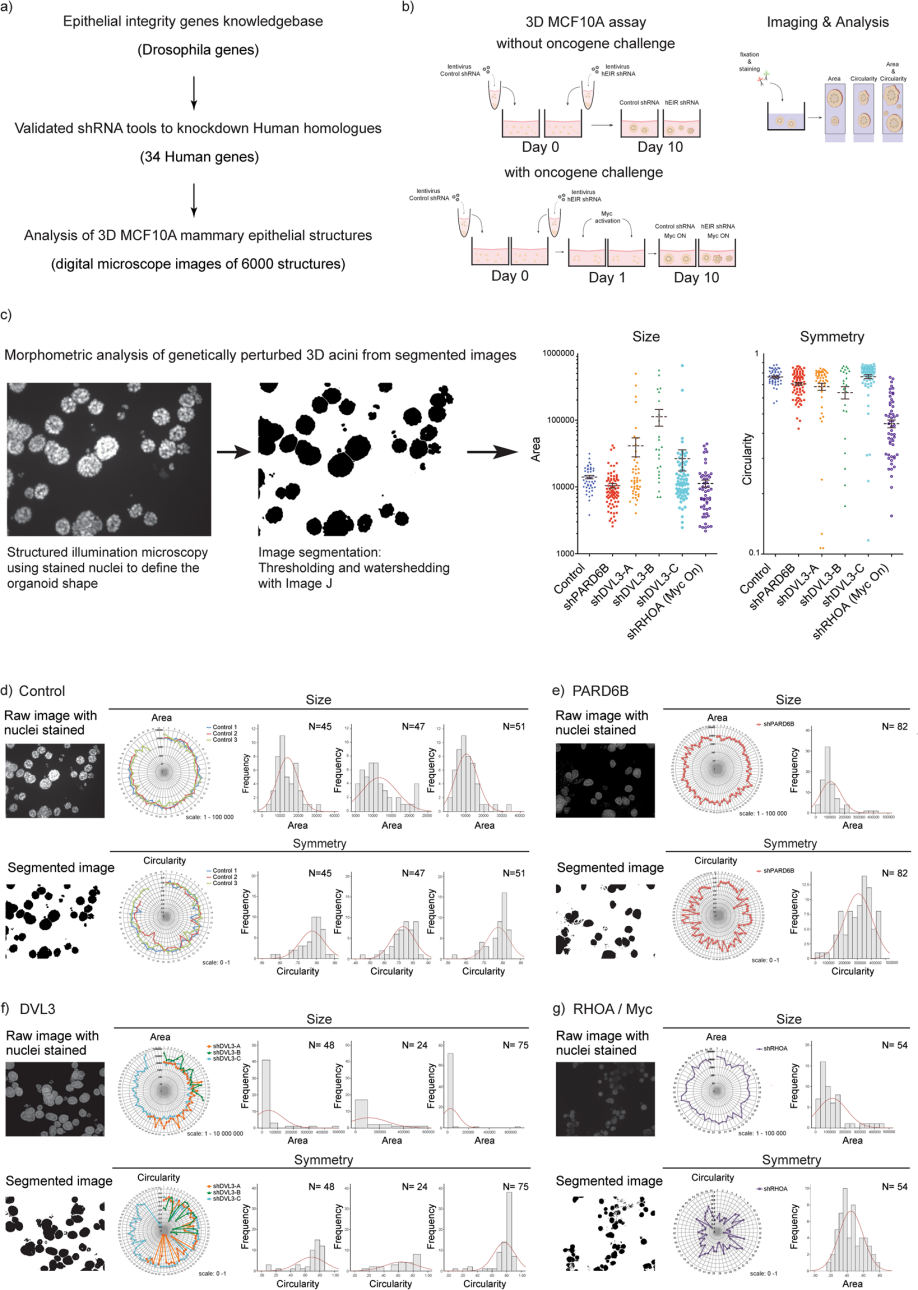
Impact of hEIR gene silencing on the phenotype of 3D MCF10A acinar structures at population level. (**a**) Outline of the shRNA screen in 3D culture. Human homologs of known Drosophila epithelial integrity regulating genes were identified and an shRNA knockdown library was designed and validated. The screen produced a databank of over 6000 images of the structures. The shRNA-induced morphological alterations were analysed in this study. (**b**) Experimental set up for analysis of shRNA perturbed 3D structures with and without oncogene challenge. The validated shRNAs were introduced in MCF10A cells expressing the conditionally active form of MYC (MycER). The shRNA expressing MCF10A acinar structures were allowed to form in Matrigel with or without active MYC for 10 days, then fixed and immunostained for morphometric analyses. In these assays, MYC activation enhanced the growth of the 3D structures and prevented the maturation-associated cell cycle exit. (**c**) Morphometric quantification of shRNA-induced phenotypic changes. Structured illumination microscopy was used to outline the shapes of the acinar structures. The shapes were captured in digital images, the original grayscale images were converted to binary images and watershed algorithm was occasionally used for segmentation. The area (proxy for size) and circularity (proxy for symmetry) values were obtained with ImageJ software. The graphs on the right show the average and distribution of the area and circularity values corresponding to example 3D acinar populations. Each dot represents a single 3D structure. (**d**–**g**) Distributions of the size and symmetry parameters of 3D acinar populations engineered to express indicated shRNAs. In each figure, the left panels show digital microscope-acquired images of the structures (as in c) and the corresponding segmented images. The radar graphs show distribution of area and circularity values obtained for the 3D acini of each population. The data distribution histograms are overlaid with the normality curve in red. (**d**) Data analysis of three independent control populations, (**e**) PARD6B silenced population, (**f**) data analysis of populations exposed to three different shRNA species targeting DVL3. (**g**) Data analysis of population exposed to active MYC together with RHOA silencing.

The morphological data examined here was derived from a recent shRNA mediated gene knockdown screen performed in a well-established mammary epithelial MCF10A 3D culture model of the mammary acinar structure. The screen was originally devised to identify genes and pathways, which play a role in regulation of acinar morphogenesis and epithelial maturation associated cell cycle exit^14^. The arrayed shRNA screen targeted Drosophila-informed human candidate genes with validated shRNAs, and identified distinct sets of genes important for establishment of proper acinar architecture and appropriately timed cell cycle restriction^14^. The shRNA effects were examined in both non-transformed and MYC oncoprotein transformed acinar structures. Altogether 34 human genes (collectively called as hEIR genes for human epithelial integrity related) could be efficiently silenced with the shRNA reagents generated in the study (Fig. 1a). The functionally validated shRNAs were lentivirally introduced into MCF10A mammary epithelial cells in an arrayed format and the cells were then directly transferred to Matrigel™, which facilitates the formation of 3D structures. The MCF10A cells used in the study contained a conditionally active MYC oncogene, which enabled analysis of the hEIR gene function under an oncogene challenge (Fig. 1b). The phenotypes developed in these cultures, with or without active MYC, were captured using structured illumination microscopy followed by digital image processing (Fig. 1c). The whole experiment produced over 6000 digital images, exposing a variety of gene knockdown induced morphological alterations^14^.

To identify suitable statistical tests for interrogation of the morphometric data, we first determined whether the 3D acinar populations were normally distributed. Examples showing area (surrogate for size) and circularity (surrogate for symmetry) value distributions within indicated acinar populations are shown in Fig. 1d–g. The silencing of indicated genes resulted in prominent phenotype alterations already observable during visual inspection of the images. Loss of PARD6B resulted in abnormally small yet symmetric acinar structures (Fig. 1e), loss of DVL3 resulted in abnormally large and asymmetric structures (Fig. 1f) whereas loss of RHOA combined with MYC (RHOA/Myc) caused widespread apoptosis leading to appearance of highly irregular structures (Fig. 1g). Shapiro-Wilk normality test of area distributions indicated that only a minority of the populations (6/55 without MYC and 4/55 with MYC) were normally distributed. Furthermore, also only a minority of circularity distributions were Gaussian (12/55 without MYC and 17/55 with MYC).

**Figure 2:**
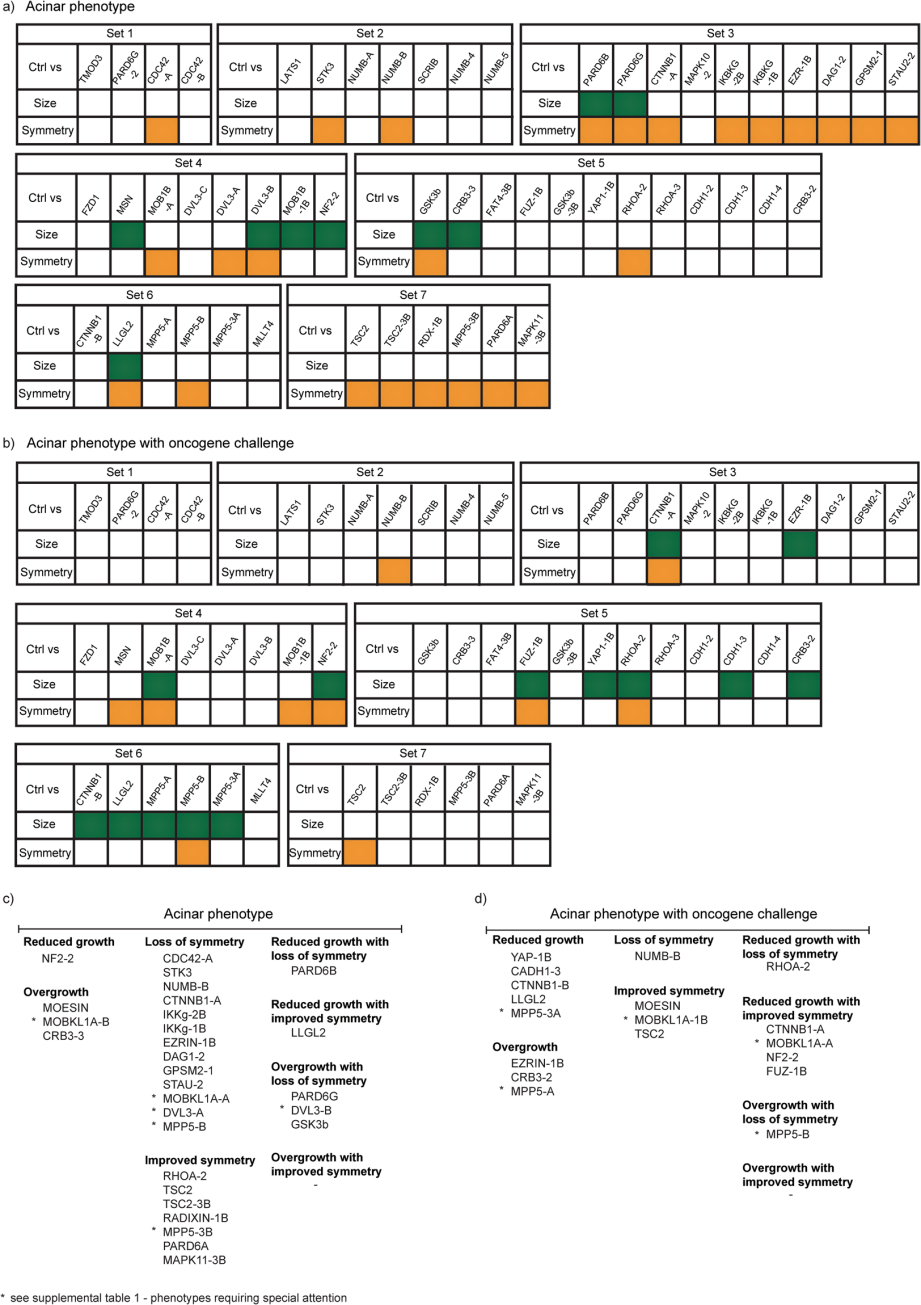
Defining phenotypically altered populations by univariate Wilcoxon Rank-Sum statistical test. (**a**) The whole shRNA screen was performed in seven independent experimental sets, with independent controls for each set. The highlighted cells denote shRNAs, which led to development of structures with significantly different size (green) and/or symmetry (yellow) as compared to control. (**b**) As in A, but the data represents acinar populations exposed to concomitant MYC activity and shRNA mediated gene silencing. (**c,d**) Categorization of the shRNAs based on their impact on the 3D morphology. List of shRNAs that showed significant impact on the morphology (**a,b**) and the subcategories that indicate the direction of the phenotype change. * highlights the phenotypes that require attention (Supplemental Table 1). Nonparametric Wilcoxon Rank Sum test; FDR < 0.05.

As a result of these population structure analyses, the statistical power of the phenotype differences was evaluated with non-parametric Wilcoxon Rank-Sum statistical test. To keep the amount of experimental work manageable, the original shRNA screen was performed as seven independent experimental sets over a time period of several years. Each experimental set included an internal shRNA control and between 4 to 12 shRNA constructs. The two-sample Wilcoxon Rank-Sum test, which is a non-parametric counterpart of the commonly used t-test, was performed for each experimental set separately. The statistical significance of the size and symmetry difference between each gene-silenced 3D acinar population and the experimental set-specific control was examined (Fig. 2a; green: significant size difference and orange: significant symmetry difference). Benjamini-Hochberg procedure was used for multiple testing correction, defining the false-discovery rate (FDR) < 0.05^19,20^. The gene-silenced 3D acinar populations grown under MYC oncogene challenge were analysed similarly and the results are shown in Fig. 2b. The analysis shows that 9 shRNA constructs, corresponding to 9 targeted genes, altered the acinar size and 25 shRNA constructs (21 genes) caused a symmetry defect (Fig. 2c). Under oncogenic MYC challenge, 14 shRNA constructs (11 genes) altered the acinar size and 10 shRNA (9 genes) led to symmetry defect (Fig. 2d). There was little overlap between the genes identified as hits in the two separate screens; with or without MYC (Figs 1b, 2c,d). These results could be explained by the fact that the examined structures were in completely different proliferative state at the time of analysis; without MYC the 3D structures were quiescent whereas chronic MYC activity prevented the cell cycle exit^14^. Surprisingly, we observed that the knockdown of numerous genes improved the symmetry of acinar structures (Fig. 2c,d). This could happen, since the control 3D MCF10A acinar structures show certain degree of heterogeneity and the average is not a perfect symmetry. Altogether, the analysis demonstrates that the shRNA mediated silencing of hEIR genes leads to morphologically altered 3D phenotypes, which are sufficiently prevalent in unselected acinar populations for statistical analysis of inter-population differences.

### Maximum mean discrepancy of the population distributions

**Figure 3:**
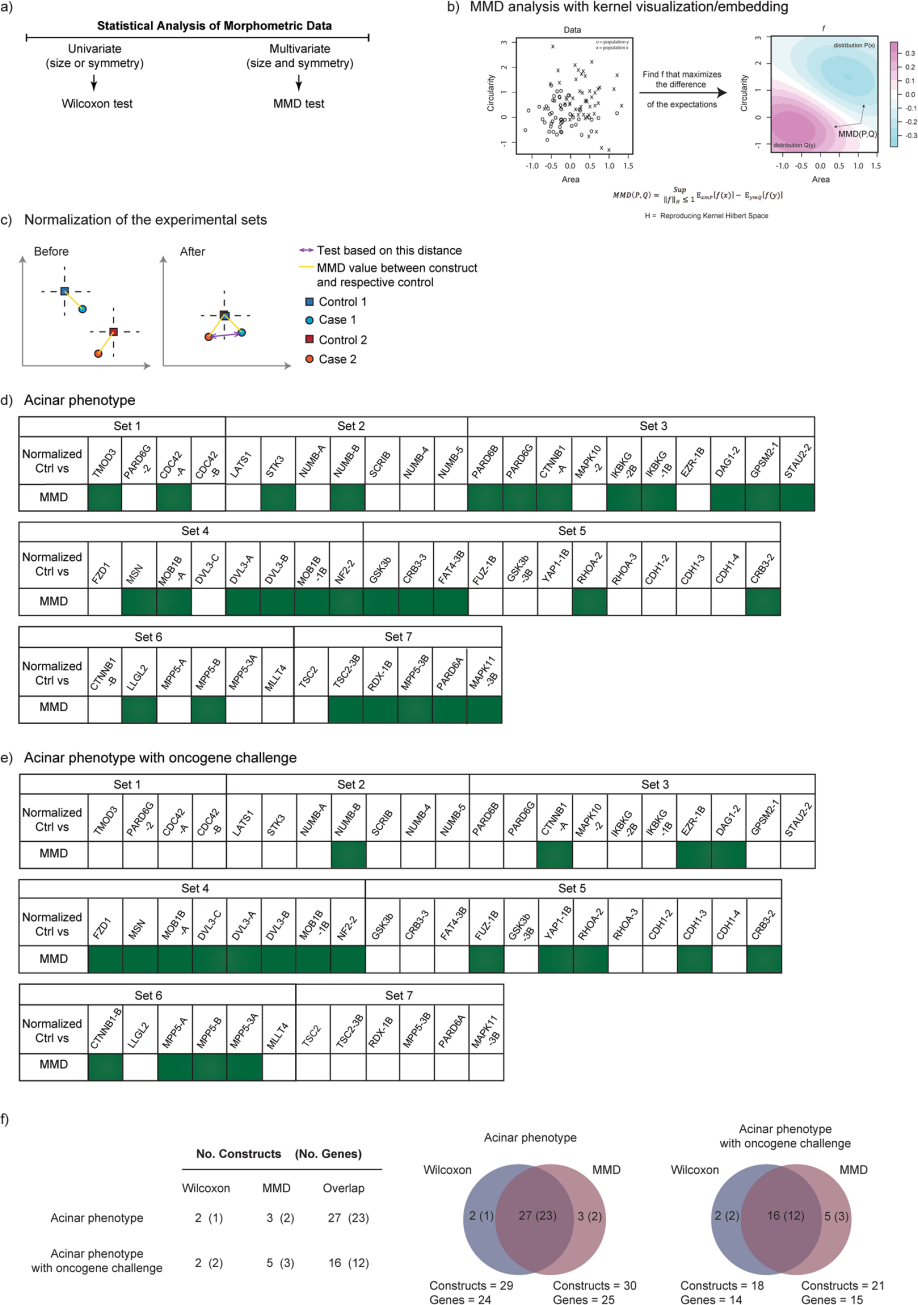
Maximum Mean Discrepancy (MMD) analysis of the data population distributions. **(a**) Diagram depicts the two statistical approaches used in this study to analyse the morphometric data. (**b**) Schematic representation of Maximum Mean Discrepancy analysis. The data are first plotted in a multivariate way, next a function that maximizes the distance between populations is applied and finally, the witness function illustrating the difference in the two populations is plotted. (**c**) Schematic representation of the normalization procedure of independent experimental sets. The populations are mean-centred using their respective control populations. The normalization procedure preserved the relative distances between the controls and the populations exposed to shRNA mediated gene silencing. (**d,e**) Summary tables showing the shRNA’s that with or without oncogene challenge resulted in populations exhibiting significantly different distribution (MMD) in comparison to normalized control (FDR < 0.05). (**f**) Table and Venn Diagram illustrate concordance between the results from Wilcoxon Rank-Sum test and MMD analysis.

The raw data examination indicated that each shRNA with discernible effect on the acinar phenotype also caused a broad distribution of the phenotype defining (size or symmetry) values within the population (Fig. 1c–g). These findings together with the notion that only a minority of the shRNA silenced populations were normally distributed were consistent with a marked overall intra-population heterogeneity. The high degree of population heterogeneity was somewhat expected finding since shRNA mediated gene silencing is not 100% penetrant. Since a change in population distribution was a consistent shRNA’s impact defining feature, we applied the maximum mean discrepancy (MMD) test to scrutinize the data population distributions in multivariate setting (Fig. 3a)^21^. The non-parametric MMD analysis (see Materials and Methods for mathematical details) is a kernel based test designed to analyse and compare probability distributions, which has recently found several applications in the analysis of multivariate data produced by molecular biology and omics approaches^17,21^. The MMD analysis is defined in terms of embedding the data probability distributions into an (implicit) infinite-dimensional feature space. Representation of the distributions in a reproducing kernel Hilbert space preserves their statistical features and allows computing distances and testing differences between them. In more practical terms, multivariate observations from two data populations are empirically compared by computing their similarities based on the chosen kernel function. For each pair of our populations, the MMD value was obtained using all the individual data-points in the populations and considering their size and symmetry values as multivariate observations (Fig. 3b). Using the MMD test, we first compared each gene-silenced population against its set-specific control with the null hypothesis that distributions of 3D acinar structures between two populations are equal against the alternative hypothesis that the distributions are different. The Benjamini-Hochberg procedure was used for multiple testing correction, defining significance at false-discovery rate (FDR) < 0.05^19,20^. To compare the 3D acinar populations across all the independent experimental sets, the control samples of each set were used for normalization. In particular, we centred the empirical embedding of the gene-silenced data populations in each experimental set by overlapping the embedding of their set-specific control, preserving the relative distances of the constructs in each independent experimental set (Fig. 3c). Altogether, 30 shRNA constructs (25 genes) were identified that resulted in significantly different population distributions from control (Fig. 3d, highlighted in green). In the presence of MYC oncogene challenge, 21 shRNA constructs (15 genes) resulted in significantly different population shape (Fig. 3e). We next examined whether the results from the two different statistical analysis (Wilcoxon Rank-Sum and MMD test) are consistent in identifying significant differences between the 3D acinar populations. The Venn diagram representations show an almost perfect overlap between the gene hits identified by the two different statistical analyses performed, thus validating the MMD approach (Fig. 3f).

### Deciphering gene function via population distribution patterns

**Figure 4:**
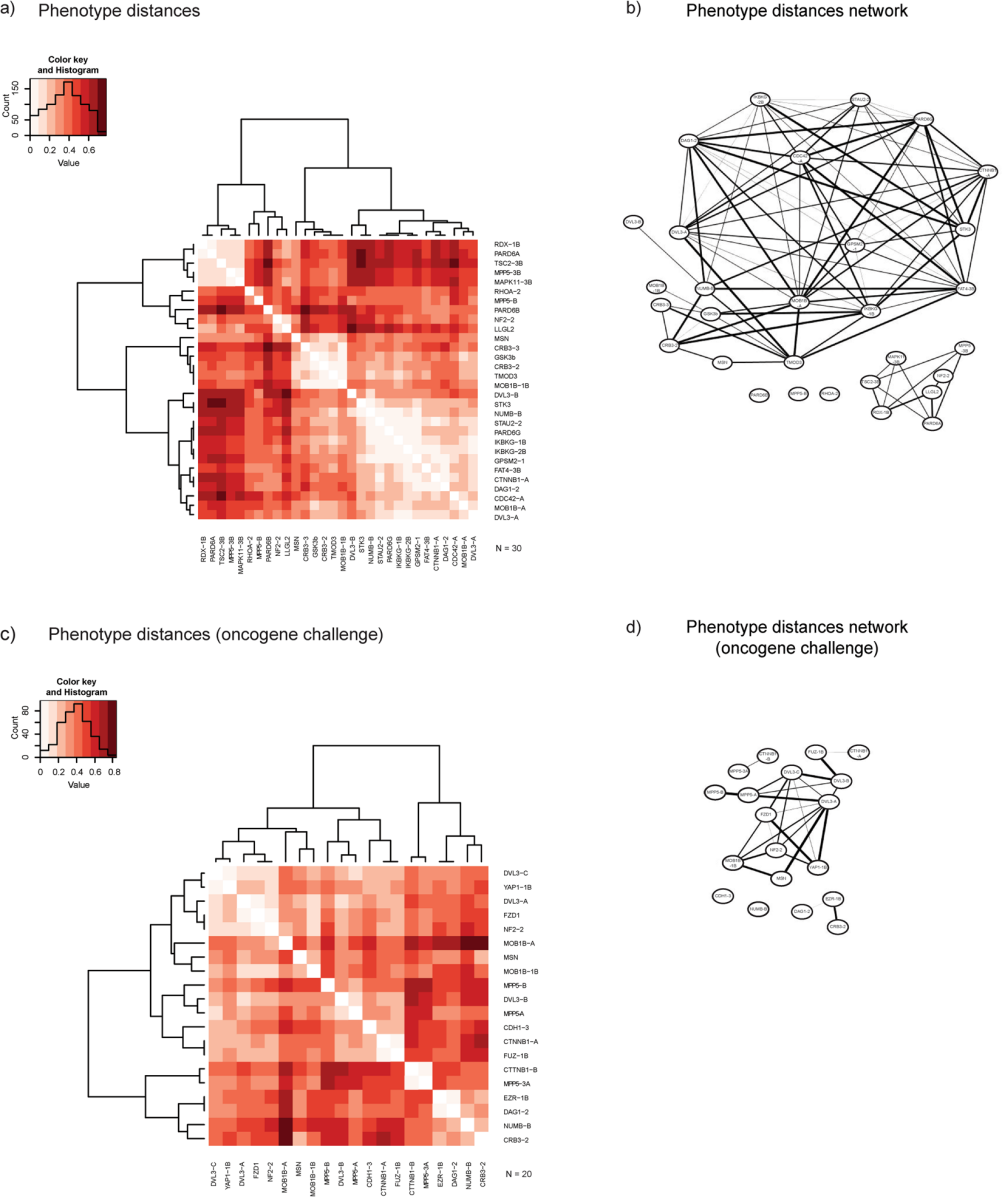
Heatmap and network representation of the MMD distances. (**a**) Heatmap representation of the populations significantly different from the control (n = 30). In heatmap, the colour gradient from light to dark red reflects an increase in the populations distance (see inset). The data was hierarchically clustered to better visualize the phenotype relationships in the data matrix. (**b**) Network representation of the 3D populations based on the experimental-set-normalized pair-wise MMD values. The network was constructed with Cytoscape software^48^; each node depicts the shRNA resulting in the phenotypic alteration and the edge (interaction) lines thickness is inversely related to the closeness of the populations (thicker lines mean longer distance). The edges were pruned by implementing the threshold of MMD ≤ 0.1. (**c**) Heatmap representation of the MYC challenged populations significantly different from the control (n = 20). (**d**) Network representation of the MYC challenged 3D populations based on the experimental-set-normalized pair-wise MMD values.

Importantly, the MMD test provided a distance metric to evaluate the relationships between different population distributions. We used both heatmap and network representation to analyse and visualize the MMD defined distances. The distance relationships of the 30 populations showing significant distances from their respective control (Benjamini-Hochberg: FDR < 0.05) are presented as heatmap in Fig. 4a and as a MMD network in Fig. 4b (network edges pruned by MMD < 0.1). Note that in the heatmaps (Fig. 4a and c) the dark red colour indicates longer distance (high MMD values) and the pale red closeness (low MMD values). In the network (Fig. 4b), each node represents shRNA silenced genes and the edges/interactions represent the MMD values (thinner line width means shorter distance). These analyses of population distribution distances exposed a number of short distance separated genes, which interestingly encode proteins of discrete signalling modules. These genes encoded, well known mediators of Wnt signalling pathway (Dishevelled/DVL; beta-catenin/CTNNB1; GSK3beta), Hippo pathway (Serine/Threonine kinase 3/STK3 (MST2); MOB kinases activator 1B/MOB1B) as well as proteins implicated in cell polarity regulation (Membrane palmitoylated protein 5/MPP5; PAR-6 family cell polarity regulators/PARD6beta and gamma; Lethal Giant Larvae Homolog 2/LLGL2; Crumbs 3/Crb3; CDC42). The same analysis and visualization methods were applied to the data populations representing simultaneous hEIR gene knockdown and MYC activation (oncogene challenge) (Fig. 4c,d). The analyses of shRNA perturbed populations grown under oncogene challenge also indicated a prominent role for Wnt and Hippo signalling pathways in growth regulation, showing that loss of these signalling modules contributes to the oncogenic MYC function in transformation of the 3D structures.

### Integrating phenotype-associated genes with proteomic data to find novel biochemical pathways

**Figure 5:**
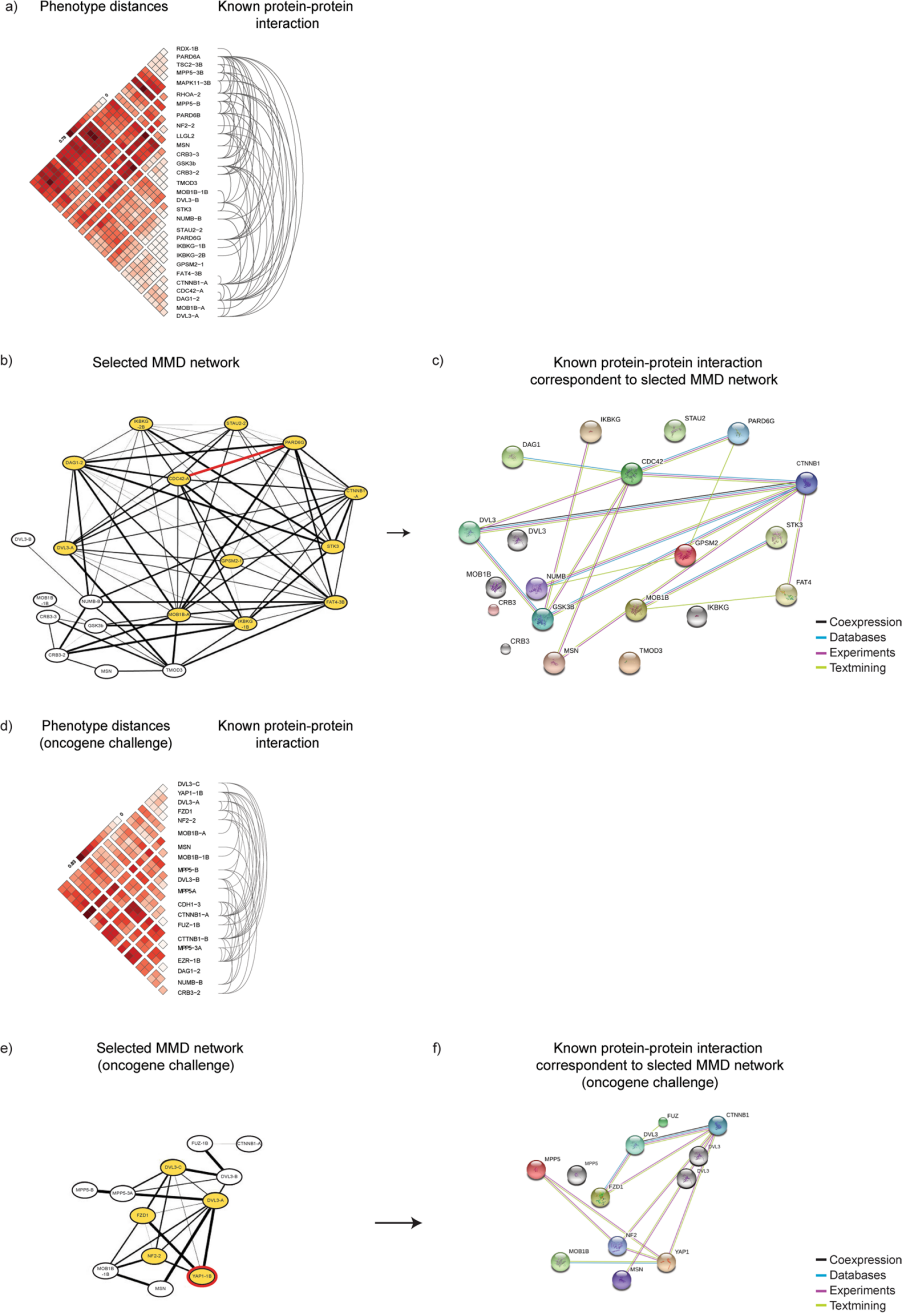
Integrated analysis of MMD-based phenotypic distances together with proteomic data predicts novel biological pathways. (**a**) Epithelial phenotype critical genes contribute to the human protein interactome. The MMD-informed genes were queried against the genes present in STRING database of known protein-protein interactions. The figure demonstrates that the 3D phenotype altering genes identified in this study have multiple interactions with each other (arcs on the right side represent interactions described in the STRING database). (**b**) Anchor interaction analysis. Anchor denotes any interaction of primary interest. The cell polarity critical PAR6-CDC42 interaction is depicted as an anchor (red) and the first neighbours of the anchor are highlighted in yellow. (**c**) STRING network representation of protein interactions corresponding to the MMD clustered genes in b). (**d–f**) The figures as in a-c but here describing populations challenged with MYC oncogene. Instead of anchor interactions, a single anchor protein YAP-1 was selected.

The MMD analysis of population distribution distances and the results from Wilcoxon test, which provided information about the direction of the morphological change, together offered data to further explore the relationship between the genes found in the present study and different biological pathways. It is well-established that the cell polarity and epithelial integrity are dynamic processes critically controlled by evolutionary conserved protein complexes, for example the PAR complex, formed of aPKC-PARD3-PARD6-CDC42 proteins, and the SCRIB complex composed of SCRIB-LLGL2-DLG^2,22–24^. To layer proteomic information on top of the current genetic and phenotypic data, we determined the presence of hEIR gene hits in the STRING database^25^. This well-known proteomic database collects information of the known and predicted protein protein interactions covering global interactome studies, experiments and published literature. The query showed that hEIR genes are well represented in the STRING database and identified large number of protein interactions among the protein products of the hEIR genes (Fig. 5a).

To integrate data from our 3D phenotype screen with proteomic data, we first explored the network representation for “anchor interactions”, which means a subjectively chosen interaction of particular interest (starting point). In the present study, we chose to focus on PARD6G-CDC42 (red line) since the physical interaction between PAR6 and the small GTPase CDC42 form a well-established and critical component of the PAR complex^26^. The PAR complex has a general and conserved role in the regulation of asymmetric cellular processes^27^, which include establishment of the apical domain of the epithelial cells and orchestration of directional cell migration and asymmetric cell division^28,29^. As a second step, we highlighted the first neighbours (yellow nodes) of both PARD6G and CDC42 to visualize the 3D acinar populations exhibiting similar distances from normalized controls as PARD6G or CDC42 (Fig. 5b). As a third and last step, we compared the phenotype network with a STRING network of protein-protein interactions (compare Fig. 5b,c). In search of potentially interesting interactions supported by functional (present study) and biochemical (STRING) data, we noticed that PARD6G-CDC42 interaction shows closeness to Dishevelled Segment Polarity Protein 3/DVL3 and beta-catenin/CTNNB1. Both DVL3 and CTNNB1 are key mediators of the WNT signalling pathway, which plays a critical role in development and carcinogenesis^30,31^. On the other hand, the data suggests phenotypic closeness between PARD6G and MOB1B but also indicate that it is currently not known if the proteins directly interact in the cells.

**Figure 6:**
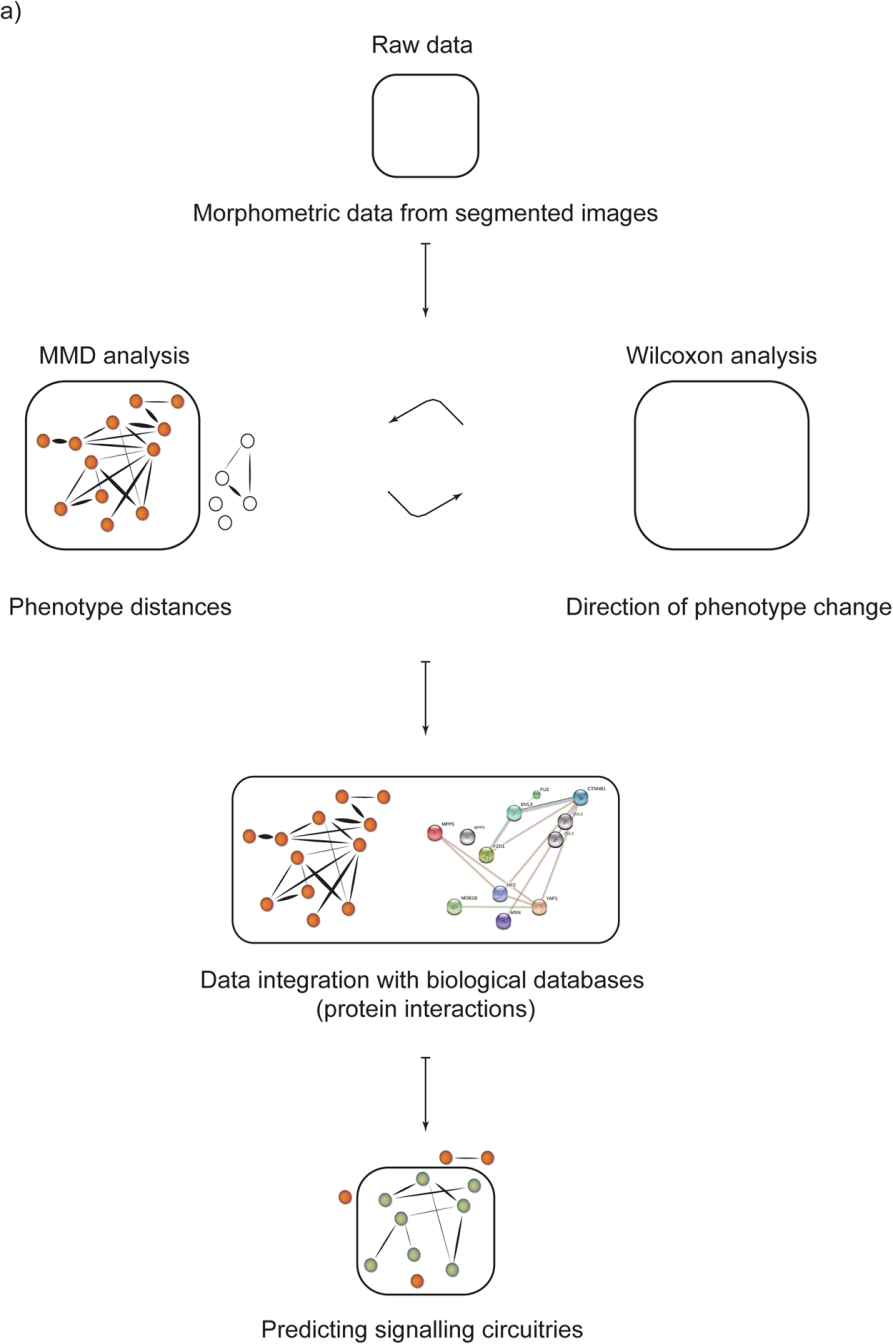
Summary of the integrated 3D phenotypic and proteomic analysis workflow. Statistical analyses and data integration steps have been described in the text.

We performed similar workflow of network analysis for the data populations representing the MYC challenged 3D structures (Fig. 5d–f). Exploration of the interactions of this smaller network did not reveal any obvious physical anchor interaction of interest. However, we considered YAP1 as an interesting anchor protein, since recent evidence indicates that the oncogenic MYC suppresses YAP/TAZ transcriptional co-activators^32^. Importantly, this MYC (up)-YAP (down) signalling axis limits the stem cell features of breast cancer cells, offering new avenues for therapeutic intervention. Our study shows that the YAP phenotype (reduced growth) is only exposed in the presence of MYC challenge, thus providing further evidence for a close signalling crosstalk between YAP and MYC. The parallel exploration of MMD and STRING networks highlighted an already known indirect interaction between YAP and NF2^33,34^. Neurofilament 2 (NF2) gene encodes a protein called Merlin, which regulates at multiple points the Hippo signalling pathway^35^. The Hippo pathway is a conserved kinase pathway, which uses YAP/TAZ to control cell proliferation and tissue growth. Furthermore, dysregulation of NF2 and Hippo pathways have significant impact on the development of human cancer^35–37^. Our data predicts that in the presence of active MYC, loss of either NF2 or YAP activity may reduce the growth potential of the transformed structures and, speculatively, result in desired therapeutic effects on tumour cells with high MYC activity. These observations highlight the potential of MMD based phenotypic analyses of genetically altered 3D structures in search of testable hypothesis relevant for understanding epithelial biology and cancer. A summary of the analysis workflow is presented in Fig. 6.

## Discussion

Reverse genetics collectively refers to approaches aiming to interrogate the gene functions via analysis of the phenotypes caused by specifically engineered or mutated gene sequences^38–40^. The reverse genetic screens are most informative when the analysed phenotypes are easy to discern, quantitatively measured and highly penetrant. These conditions are often met in screens using single cells, such as yeast cells or simple genetically tractable organisms such as *C. elegans*, Drosophila or Zebrafish^41,42^. In mammalian systems, the complexity of tissue structures and long life-span of the organisms have long restricted genetic screens to simple 2D petri-dish based cell cultures, which are highly reductionist cell systems with respect to the highly organized 3D tissue structures of epithelial cells *in vivo*. However, the recent emerge of mammalian epithelial 3D cultures, which preserve or mimic tissue cohesion, cell-cell and cell-matrix interactions as well as many physical variables of the microenvironment, have offered new possibilities to broaden our understanding of the context-dependent regulation of individual cell functions^43^. Nonetheless, reverse genetic screens in 3D culture present many unanticipated technical hurdles not only for the experimental design but also for data analysis.

In the present study, we introduce a novel statistical framework for the analysis of 3D culture phenotype data. We accessed the data originating from an shRNA screen in MCF10A 3D culture, which employed 53 validated shRNAs designed to target 34 genes. Over 6000 digital images of MCF10A structures were analyzed. The multivariate statistical framework axiomatically differs from the previous analysis by Marques *et al*.^14^, which used averages and fold-changes separately for the phenotypic traits (size and symmetry) as the statistics to investigate the data. In the present study, following the investigation of the shape of the statistical distributions of the traits, and their evident non-normality, we based the analysis on non-parametric statistics, the Wilcoxon rank-sum test and the maximum mean discrepancy (MMD) statistic. MMD is a state-of-the-art statistical method for two-sample testing based on an elegant mathematical theory^21^ and is widely applicable to different types of data (including structured data, such as strings and graphs), providing extensibility^18,21^. MMD considers the distribution of all the measured traits simultaneously to quantify the differences in the populations, and, it provides a metric space for the population distances. The test has previously been used in biological analysis, for example, to integrate biomarker data^18^, to minimize measurements variations when data are acquired in different experimental replicates and/or batches^44^, and to identify similar gene expression patterns in different experimental conditions^17^. In the current study, the results from MMD analysis agreed well with the traditional univariate Wilcoxon Rank-Sum test, which is a non-parametric equivalent of the paired t-test. These statistical approaches, when used in integrated manner, enabled us to identify the most significant phenotypic changes, organize them according to the direction of the change (small, large, loss of symmetry, improved symmetry), cluster closely related distributions and superimpose these clusters with global data on biomolecular interactions. This workflow of analyses, which is summarized in Fig. 6, predicted novel cell signaling pathways around PARD6-CDC42 cell polarity regulating complex. Furthermore, the integrated analysis suggested Hippo pathway effectors being essential for MYC-dependent deregulation of the 3D structures growth.

It is important to note, that even though the present study employed pre-validated shRNA species, there are number of remaining error sources to be considered when interpreting the data. For example, RNA interference mechanism inherently produces gene knockdown with non-congruous cell-to-cell efficacy rather than complete gene knockout^45^. Furthermore, the 3D structures develop from one cell and occasionally through fusions of neighbouring structures^14^. Thus, only a certain fraction of the shRNA-transduced individual 3D acini will exhibit the maximal phenotypic change. In addition, many phenotypic effects were observed only with a single shRNA species and the primary screen data represent a single experiment rather than biological replicates. Due to these fallibilities, which apply for any shRNA screen in 3D culture, the phenotype distance networks and related biochemical data, STRING protein-protein interactions provided here as an example, should be interpreted with caution. Nonetheless, the Wilcoxon-MMD statistical framework is expected to have many practical applications. It provides a set of tools for hypothesis generation on the basis of the primary screening data and, we believe that there is an increasing need for such tools especially, in the analysis of large datasets. Completion of a screen always requires further validation of the hits, but especially in the case of phenotype based screens, it is not always evident what to prioritize in hit picking. The hits are often prioritized on a subjective basis (interesting gene or pathway) or according to the strength of the phenotypic change. However, these approaches are prone to confirmation bias. The statistical framework presented in this study, which integrates Wilcoxon rank-sum and MMD tests, serves as a versatile solution for hypothesis-guided hit selection. In the presented solution, one can choose the first gene hit or gene interaction on a subjective basis and, subsequently, select neighborhood genes on the grounds of putative phenotype closeness. The hypothesis-guided hit picking does not exclude other approaches, but generates opportunity to identify novel phenotype impacting genetic interactions around the original gene of interest. The presented statistical system for 3D culture data analysis is generalizable and can be applied to deal with the large datasets from high-throughput analysis of miniaturized 3D cultures. However, it is stressed again, that the primary screen derived hypothetical interactions will have to be validated with appropriate laboratory analyses. In summary, the solution presented in our study is expected to facilitate organization and analysis of the data from genetic screens that employ 3D culture systems. Furthermore, the Wilcoxon-MMD statistical framework provides an explorative platform that allows integration of phenotypic data with different biological knowledgebase for generation of new testable hypotheses on epithelial pathway functions.

## Material and Methods

### Construction of shRNA library, cell biological experiments and data acquisition

The shRNA library targeting the Human Epithelial Integrity Regulator genes (hEIR), experimental methods related to the MCF10A 3D culture and image acquisition procedures are described in Marques (2016)^14^.

### Statistical tests

Kolmogorov-Smirnov and Shapiro-Wilk normality tests^46^ were performed using SPSSStatistics Ver. 22.0.0.1.

The non–parametric Wilcoxon rank sum test was used to define significant size and symmetry differences within the independent experimental sets followed by multiple test correction using the Benjamini Hochberg (FDR) method. The threshold FDR < 0.05 was used to define hits. All tests were performed using the R software for statistical computing^47^.

### MMD analysis of the 3D population distributions

#### Preprocessing

Observed morphometric parameters, area and circularity, were log-transformed and logit-transformed respectively. Transformed values were then scaled to have unit variance over the aggregate of all control and knockdown samples. The raw data processing was performed independently for the samples with or without oncogene challenge.

### Data analysis

A non-parametric two-sample statistical test was adopted to calculate the maximum mean discrepancy (MMD) between two data populations of interest. The MMD statistic developed by Gretton 2012^21^ restricts the function choice to a certain function class (unit ball in a reproducing kernel Hilbert space (RKHS)) and allows estimating MMD as averages of kernel evaluations over all the observed samples.

To test the difference between two data populations, the following MMD statistics was applied:

1 $$MMD=\begin{array}{c}sup\\ f\end{array}{E}_{x}[f(x)]-{E}_{Y}[f(y)]=\parallel {\mu }_{x}-{\mu }_{y}\parallel ,$$

where x and y represent the populations distributions and **f** is defined to maximize the difference between them. The statistic is the distance between the mean embedding ${\mu }_{x}$ and ${\mu }_{y}$ of the two population distributions in the RKHS (they are expectations of the kernel feature space over the distributions). We used the MMD with Gaussian kernel where the length-scale is chosen with the median distance heuristic over aggregate samples. The null distribution for the hypothesis that x and y have the same distribution is generated by simulation (permutation testing) and the p-value is estimated from M permutations as:

2 $$p-value=\frac{{\sum }_{i}^{M}I(MM{D}_{i}^{2} > MM{D}^{2})+1}{M+1},$$

where $MM{D}_{i}^{2}$, i = 1, …, M are the permutation statistics and MMD^2^ is the observed test statistic.

Since multiple statistical tests were performed, a false discovery rate (FDR) control defining a p-value cut-off was applied as follow:

3 $$FDR=E[\frac{T}{T+F}],$$

where T is the number of true discoveries and F is the number of false discoveries (T + F is the total number of tests declared significant). A test is defined as interesting knockdowns if the FDR corrected p-value is <0.05^19^.

### MMD distance based network construction

To normalize MMD estimates across the independent experimental sets, the MMD statistics was extended as:

4 $$\begin{array}{rcl}MMD & = & \begin{array}{c}sup\\ f\end{array}({E}_{x}[f(x)]-{E}_{A}[f(a)])\mbox{--}({E}_{y}[f(y)]-{E}_{B}[f(b)])\\  & = & \parallel ({\mu }_{x}-{\mu }_{A})-({\mu }_{Y}-{\mu }_{B})\parallel \end{array},$$

where A and B are the respective control populations for X and Y. If X and Y are from the same experimental set, ${\mu }_{A}={\mu }_{B}$ and the MMD statistic simplifies to the previous definition. Otherwise, the independent experiments correction will mean-centre the two samples using their respective control means in the RKHS. The networks were built greedily by connecting the two knockdowns with the smallest MMD and so on until a set threshold. After computing the MMD values for each pair of populations, we clustered the populations based on the MMD values using hierarchical clustering with Ward’s criterion (R function hclust with ward.D method). Using Cytoscape software^48^, we produced pruned heatmaps and networks, where we only include populations that show different phenotype from the controls (network edges pruned by MMD ≤ 0.1). To integrate our population distances to known protein-protein interactions we used the STRINGdb (version 10) with confidence level 0.7 and source of evidence set as in^49^.

## Electronic supplementary material

Supplemental Table 1
