# Supplementary material for: Phenotype-driven identification of epithelial signalling clusters
Source: Sci Rep. 2018 Mar 5;8:4034. doi: 10.1038/s41598-018-22293-x (PMC5838230; doi:10.1038/s41598-018-22293-x)
Supplement: Supplementary file 1 — Supplemental Table 1 [file 41598_2018_22293_MOESM1_ESM.pdf]

Supplementary information for:

# Phenotype-driven identification of epithelial signalling clusters

**Elsa Marques[1], Tomi Peltola[2], Samuel Kaski[2], Juha Klefström[1]***

[1] Cancer Cell Circuitry Laboratory, Research Programs Unit / Translational Cancer Biology & Medicum, University of Helsinki, P.O Box63 (street address: Haartmaninkatu 8), 00014 University of Helsinki, Finland

[2] Helsinki Institute for Information Technology HIIT, Department of Computer Science, Aalto University, PO BOX 15400, FI-00076 Aalto, Finland

**The PDF file includes:**

**Supplemental Table 1.** Table of hEIR phenotypes requiring special attention.

Supplemental Table 1 - ***Table of hEIR phenotypes requiring special attention***

| ***Gene name*** | ***Note*** | ***Results / Figure*** | ***Explanation*** | ***References*** | ***Recommended actions*** |
|---|---|---|---|---|---|
| MPP5 | Inconsistent phenotypes | Figure 2 | Knockdown of MPP5 yelds unexpected results across experiments (with and without oncogene challenge).<br>Phenotypes corresponding to 3 different shRNA fell into different categories:<br>- without oncogene challenge:<br>Loss of symmetry; Improved symmetry<br>- with oncogene challenge:<br>Reduced growth; Overgrowth with loss of symmetry; Overgrowth | NA | Experiments should be repeated with a new set of validated shRNA |
| MOBKL1A | Inconsistent phenotypes | Figure 2 | Knockdown of MOBKL1A yelds unexpected results across experiments (with and without oncogene challenge).<br>Phenotypes corresponding to 2 different shRNA fell into different categories:<br>- without oncogene challenge:<br>Loss of symmetry; Overgrowth<br>- with oncogene challenge:<br>Improved symmetry; Reduced growth with improved symmetry | NA | Experiments should be repeated with a new set of validated shRNA |
| DVL3 | Inconsistent phenotypes | Figure 2c | Knockdown of DVL3 yelds unexpected results across experiments without oncogene challenge.<br>Phenotypes corresponding to 3 different shRNA fell into different categories:<br>- without oncogene challenge:<br>Loss of symmetry; Overgrowth with loss of symmetry | NA | Experiments should be repeated with a new set of validated shRNA |
